# Health and ageing in Nairobi’s informal settlements-evidence from the International Network for the Demographic Evaluation of Populations and Their Health (INDEPTH): a cross sectional study

**DOI:** 10.1186/s12889-015-2556-x

**Published:** 2015-12-11

**Authors:** Boniface Wilunda, Nawi Ng, Jennifer Stewart Williams

**Affiliations:** United Nations Office at Nairobi (UNON), UN Gigiri Office Complex, Block X, P.O Box 30218–00100, Nairobi, Kenya; Unit of Epidemiology and Global Health, Department of Public Health and Clinical Medicine, Faculty of Medicine, Umeå University, Umeå, Sweden; Centre for Demographic and Ageing Research, Umeå University, Umeå, Sweden

**Keywords:** Functional health, Domains of health, Quality of life, Informal settlements, Slums, Ageing, Aging, Nairobi, Kenya, Africa

## Abstract

**Background:**

Much of the focus on population ageing has been in high-income counties. Relatively less attention is given to the world’s poorest region, Sub-Saharan Africa (SSA) where children and adolescents still comprise a high proportion of the population. Yet the number of adults aged 60-plus in SSA is already twice that in northern Europe. In addition, SSA is experiencing massive rural to urban migration with consequent expansion of informal urban settlements, or slums, whose health problems are usually unrecognised and not addressed. This study aims to improve understanding of functional health and well-being in older adult slum-dwellers in Nairobi (Kenya).

**Methods:**

The study sample comprised men and women, aged 50 years and over, living in Korogocho and Viwandani, Nairobi, Kenya (*n* = 1,878). Data from the International Network for the Demographic Evaluation of Populations and Their Health (INDEPTH) and the WHO Study on global AGEing and adult health (SAGE Wave 1) were analysed. The prevalence of poor self-reported quality of life (QoL) and difficulties in domain-specific function is estimated by age and sex. Logistic regression investigates associations between difficulties in the domains of function and poor QoL, adjusting for age, sex and socio-demographic factors. Statistical significance is set at P<0.05.

**Results:**

Women reported poorer QoL and greater functional difficulties than men in all domains except self-care. In the multivariable logistic regression the odds of poor QoL among respondents with problems or difficulties in relation to affect (OR = 7.0; 95%CI = 3.0-16.0), pain/discomfort (OR = 3.6; 95%CI = 2.3-5.8), cognition (OR = 1.8; 95 %CI = 1.2-2.9) and mobility (OR = 1.8; 95%CI = 1.1-2.8) were statistically significant.

**Conclusions:**

The findings underscore differences in the domains of functional health that encapsulate women and men’s capacities to perform regular activities and the impact of poor functioning on QoL. Investing in the health and QoL of older people in SSA will be crucial in helping the region to realise key development goals and in opening opportunities for improved health outcomes and sustainable economic development.

## Background

Population ageing is a trend that started in developed countries where advances in medicines and technology, and economic development, have substantially reduced morbidity and delayed mortality. Between 1950 and 2010, the proportion of people aged 60 and over in the developed world increased from 12 to 22 %. This percentage is projected to reach 32 % by 2050. The top three countries with the highest population shares comprising people aged 60 and over in 2011 were Japan (3 1%), Italy (27 %) and Germany (26 %). In 2050, this ranking is  expected to be Japan (42 %), Portugal (40 %) and Bosnia and Herzegovina, also 4 % [1]. In low-and middle-income countries (LMICs), the share of people aged 60 and over rose from just 6 % in 1950 to 9 % today, and is expected to reach 20 % by 2050. However what is of significance for governments world-wide, is that between 2010 and 2050, the proportion of people who are aged 60 and over is expected to increase from 800 million to 2 billion, with 1.2 billion of these older adults living in LMICs [1, 2].

Ageing is as an important issue in LMICs as it is in developed countries. Yet much of the focus on population ageing has been in high-income countries. Relatively less attention has been given to ageing in the poorest region in the world - Sub-Saharan Africa (SSA) where children and adolescents still comprise a high proportion of the population. In 2011 over 40 % of people in SSA were children aged under 15 years, compared with 17 % in the European region and 25 % in the Americas [3]. However the number of people aged 60 years and over in SSA is already twice that of older adults in northern Europe. Recent projections estimate that the portion of the population aged 60 and over in SSA will increase from 46 million in 2015 to 157 million by 2050 [1, 4].

There are over 45 developing countries in SSA. The population of these countries as a whole is over 973 million and life expectancy at birth is 57 years. Kenya is situated on the eastern part of the African continent, and is one of the more populous developing countries in SSA, with a population of almost 45 million and life expectancy at birth of 62 years [5, 6]. The annual population growth rate in Kenya was 2.6 % between 2001 and 2011, compared with 2.4 % in SSA. In contrast, comparative rates of population growth in the European and South-East Asian Regions were 0.3 and 1.4 % respectively [7, 8]. In Kenya, people aged 60 years and over constitute about 4.7 % of the total population and this figure is projected to increase to 10.4 and 26.4 % by 2050 and 2100 respectively [8].

Apart from these major demographic changes, SSA is also experiencing massive rural to urban migration. This has resulted in the formation and expansion of informal urban settlements or slums [9, 10]. About one third of the urban population in developing countries live in slums and this proportion is 62 % in SSA [11]. It is expected that by 2030, more than half of the total population of SSA will live in urban slums [12, 13]. Nairobi, the capital of Kenya, will have over two thirds of its inhabitants occupying informal urban settlements [14].

In SSA countries, older people have traditionally been respected for their wisdom and roles they play as heads of families. However in recent years, economic and social change and patterns of migration, have weakened traditional social values and networks that provide care in later life [9, 15]. In Kenya, older people have been neglected in many of the country’s policies and programmes but this is starting to change [9]. One of the Kenyan government’s main population and development objectives is aimed at improving and promoting public awareness of the importance of healthy ageing and QoL [16]. Yet older adults living in urban slums exhibit poorer health and QoL compared with non-slum dwellers or their rural counterparts [10, 12]. Studies on the health and well-being of older adults in informal settlements are necessary to fill the evidence gap in this important area.

In addition to the presence or absence of chronic illnesses that require ongoing healthcare treatment and management [17, 18], older adults’ QoL is related to their capacity to function, physically, cognitively and emotionally on an everyday level. Functional health comprises a constellation of domains in which older adults may experience deficits, for example, in vision, mobility and cognition, that impact on their QoL. Yet although overall summary measures of functional health are important, they can overlook specific aspects and mask offsetting effects. For example, a person who scores well on cognition but poorly on pain management may have a good overall health rating. In high-income countries, there has been growing public health interest directed at improving understanding of the key components of everyday functioning so that appropriate policies and services can be developed, implemented and evaluated. This perspective is also important for low-income countries where populations are ageing rapidly and many older adults are experiencing major social and economic transitions, including migration, with little being known about their functional health and QoL [19, 20].

The World Health Organization (WHO) has been instrumental in promoting understanding of individuals' self-assessed ‘health state’ in terms of functioning and disability [21]. A common interpretation is the view that the health state reported by individuals consists of functioning in spheres or domains [22]. WHO has developed and tested eight core domains of functional health - mobility; self-care; pain/discomfort; cognition; interpersonal activities; vision; sleep/energy, and affect – which are widely accepted as being of fundamental importance to all human beings, irrespective of age, culture, social or socioeconomic circumstances [21, 23, 24].

This study describes domain-specific functioning and QoL in older adult slum-dwellers in Nairobi, Kenya [10, 25]. The objectives are to compare the prevalence of the WHO’s self-assessed functional difficulties and poor self-reported QoL in men and women aged 50 and over, and describe associations between multiple attributes of daily function and QoL, adjusting for age, sex and socio-demographic factors.

## Methods

### Study area and participants

The African Population and Health Research Centre (APHRC) is implementing the longitudinal Nairobi Urban Health and Demographic Surveillance System (NUHDSS) study as part of a larger health life course study in Kenya’s capital city Nairobi [26]. See http://www.indepth-network.org/Profiles/Nairobi%20UHDSS.pdf. The NUHDSS is a member of the International Network for the Demographic Evaluation of Populations and Their Health (INDEPTH) (http://www.indepth-network.org), which comprises an international network of 52 health and demographic surveillance systems (HDSS) in 20 countries in Africa, Asia, Oceania and Central America. The NUHDSS covers large areas of Korogocho and Viwandani informal settlements or slums, located five to ten kilometres from Nairobi city centre. Compared to formal settlements, housing in informal settlements is without official approval e.g. from local authorities. Informal settlements are therefore characterized by inadequate infrustructure, poor access to basic services such as sanitation, water and electricity, and unsuitable living conditions. Since January 2003, data on core demographic events (births, deaths, migration) have been collected and updated every 4 months as part of routine NUHDSS rounds [10].

### Data collection

In 2006–2007, the INDEPTH and WHO collaboration implemented the short-form of the individual INDEPTH-WHO SAGE (Study on Global Ageing and Adult Health) questionnaire in the NUHDSS. Details of INDEPTH-WHO SAGE are reported elsewhere. The HDSS collected data from the entire population of adults aged 50 years and over in the NUHDSS [27]. Data were collected through household visits and interviews. All interviewers were required to have had a minimum education of 12 years of schooling, reside in the NUHDSS area and have undergone training covering a 5-day period followed by 2 days of field testing. Each group of five interviewers was supervised by a team leader who conducted random spot checks on the accuracy of the information being collected and offered additional training as necessary [10]. Data were entered and cleaned at the field station in Nairobi and sent to Umeå University (Sweden) for further cleaning, imputation of missing data and harmonisation of data sets from all HDSS sites [27]. A dataset for the Nairobi NUHDSS was provided by WHO-INDEPTH and used in these analyses.

### Variables

The independent variables are discrete measures of functional health in the eight WHO domains of functioning - mobility; self-care; pain and discomfort; cognition; interpersonal activities; vision; sleep/energy, and affect. They refer to the difficulties/problems faced in performing activities in each functional domain during the preceding 30 days. Responses were ranked according to a five-point Likert scale: none vs. mild vs. moderate vs. severe vs. extreme/cannot do [22]. Where responses to questions were either none, mild or moderate, respondents were classified as having ‘good’ functional health, and where responses were either severe and extreme or cannot do, respondents were classified as having ‘poor’ functional health i.e. difficulties and problems in performing associated activities [22].

The measurement of QoL is captured by the WHO Quality of Life instrument (WHOQoL) which has been used widely in low-income countries [28] and was developed by an international panel of researchers over a 12 year period. This development involved using a unique set of study protocols to ensure cross-cultural applicability and a high degree of functional and metric equivalence across different cultures and settings [19]. The WHOQoL instrument is widely used to study older adults’ QoL in LMICs [27]. Quality of life refers to subjective well-being or how a person feels [19]. Functioning, on the other hand, refers to objective performance in a given domain or capacity to undertake everyday tasks within the constraints or otherwise of the domain e.g. experiencing pain or having poor vision.

The WHOQoL instrument measures the individual’s subjective state using eight items asking about levels of satisfaction with various aspects of life. Self-reported responses to these questions are based on a five-point Likert scale where ‘1’ indicates high satisfaction and ‘5’ indicates low satisfaction. The summary index of QoL is measured on a scale of 0 (worst QoL) to 100 (best QoL). Scores were normally distributed and dichotomised using a median cut-point designating good vs. poor QoL [10]. A complete set of study instruments and questions used is available at http://www.who.int/healthinfo/sage/indepth/en/.

Socio-demographic and economic variables are: age-group (50–59 years, 60–69 years, 70–79 years, 80-plus years); sex (men, women); education (more than 6 years, less than 6 years, no formal education); marital status (in current partnership, now single); living arrangement (living with other(s), living alone); household size (1–2 members, 2–4 members, 5 members and over), and wealth quintiles as measure of socioeconomic status. A random-effects probit model was used to estimate wealth levels based on ownership of household assets such as chairs, tables and access to electricity [24, 29]. Wealth quintile 1 represents the poorest fifth of each country’s respondents in terms of household asset-based wealth, and quintile 5 represents the highest (wealthiest fifth).

### Statistical methods

The WHO-INDEPTH SAGE data in Kenya are weighted (using sampling weights provided by WHO-INDEPTH) to the Nairobi NUHDSS population (2007) to account for survey design and adjust for age and sex differences. All variables are categorical. Descriptive statistics are presented as weighted proportions (%s) and weighted estimates (n_s_) by socio-demographic characteristics, the domains of function and QoL [9, 10]. A breakdown of the prevalence of domain-specific functional difficulties is given by sex and age groups. Chi-squared tests of significance are used to compare sex differences in socio-demographics, the domains of function and QoL.

Multivariable logistic regression tested associations between the domain-specific functions as binary independent variables (good function vs. poor function), and QoL (good QoL vs. poor QoL). Eight separate logistic regressions were undertaken with each domain as the single independent variable and QoL as the dependent variable while adjusting for socio-demographic factors as possible confounders. We also conducted logistic regression to test the association between all the domains and QoL in the same regression, while adjusting for possible socio-demographic confounders. Socio-demographic covariates used to adjust for possible confounding were: age, sex, education, marital status, living arrangement, wealth quintiles and household size [10]. Multicollinearity was tested using the Variance Inflation Factor (VIF).

Odds ratios and 95 % confidence intervals are reported. STATA Version 12 (StataCorp, 2011) was the software used for statistical analyses. The plot was constructed in R.

### Ethics approval

Ethical approval was granted from the WHO ethical review committee as part of the SAGE study. In Kenya, ethical approval was granted by the Kenya Medical Research Institute (KEMRI) Ethics Committee. Informed consent was obtained from respondents prior to data collection [10].

## Results

Residents of the NUHDSS, aged 50 years and over as of 1^st^ of October 2006, were visited. A cleaned data set of complete cases and population weights for the 2007 Nairobi NUHDSS population in Korogocho and Viwandani aged 50-plus years was made available (*N* = 1,991). The weighted population analysed here was 1,878.

Table 1 shows the background characteristics of men (*n* = 1,260) and women (*n* = 618) in the weighted study sample. Women tended to be older, less educated, single and living in larger households. Men were more likely than women to live alone. The prevalence of poor QoL was significantly higher in women than men. With the exception of the self-care domain, sex differences in the domains were statistically significant (*p* < 0.05) with women reporting greater functional difficulties than men.

**Table 1:** Background characteristics of respondents by sex, among adults aged 50 and over NUHDSS Korogocho and Viwandani Nairobi City, Kenya, 2006–7

Variables	Men	Women	Total	*p*-value
Total n(%)	1260 (67.1 %)	618 (32.9 %)	1878 (100 %)	
Age-group n(%)				*p* < 0.001
50–59 years	909 (72.2)	359 (58.0)	1271 (67.6)	
60–69 years	250 (20.0)	156 (25.2)	404 (21.6)	
70–79 years	72 (5.7)	62 (10.1)	133 (7.1)	
80-plus years	29 (2.3)	41 (6.7)	70 (3.7)	
Education n(%)				*p* < 0.001
More than 6 years	252 (20.0)	37 (6.0)	289 (15.4)	
Primary <6 years	785 (62.3)	286 (46.3)	1071 (57.0)	
No formal education	223 (17.7)	295 (47.7)	518 (27.6)	
Marital status n(%)				*p* < 0.001
In current partnership	1128 (89.5)	185 (30.0)	1313 (69.9)	
Now single	132 (10.5)	433 (70.0)	565 (30.1)	
Living arrangement n(%)				*P* < 0.01
Living with other(s)	922 (73.2)	499 (80.8)	1422 (75.7)	
Living alone	338 (26.8)	119 (19.2)	456 (24.3)	
Wealth Quintiles n(%)				*p* < 0.001
Poorest quintile	359 (28.5)	101 (16.3)	460 (24.5)	
2nd quintile	145 (11.5)	137 (22.2)	282 (15.0)	
3rd quintile	241 (19.1)	142 (22.9)	383 (20.4)	
4th quintile	243 (19.3)	151 (24.6)	394 (21.0)	
Least poorest quintile	272 (21.6)	87 (14.1)	359 (19.1)	
Household size n(%)				*P* < 0.01
1–2 members	570 (45.2)	226 (36.8)	797 (42.4)	
3–4 members	252 (20.0)	158 (25.5)	409 (21.8)	
5 members and over	438 (34.8)	234 (37.8)	672 (35.8)	
Domains of functional health % of people reported difficulties in each domain below				
Mobility	5.6	17.4	9.5	*p* < 0.001
Self-care	2.3	3.6	2.8	*p* = 0.11
Pain/Discomfort	5.7	17.1	9.6	*p* < 0.001
Cognition	6.1	12.7	8.3	*p* < 0.001
Interpersonal activities	1.5	3.3	2.1	*p* = 0.01
Sleep/Energy	3.9	10.8	6.2	*p* < 0.001
Affect	2.6	8.5	4.6	*p* < 0.001
Vision	3.3	7.1	4.5	*p* = 0.001
QoL				
% with poor QoL	36.4	55.2	42.6	*p* < 0.001

Note: All analyses weighted to the Nairobi NUHDSS population 2007. *P*-values reported for chi-squared tests of significance comparing men and women

The prevalence of poor function was higher in older age (Table 2). In the oldest age group (80-plus) men and women reported similar levels of functional difficulties. However in the 50–59, 60–69 and 70–79 year age groups, women reported significantly greater difficulties than men (*p* < 0.05) for mobility and pain/discomfort. In the 50–59 and 60–69 age groups differences between men and women were statistically significant *(p* < 0.05) for sleep/energy and affect with women also reporting greater difficulties/problems than men.

**Table 2:** Prevalence (%) of poor functional health in domains by age and sex, adults aged 50 and over NUHDSS Korogocho and Viwandani Nairobi City, Kenya, 2006–7

	50–59 years			60–69 years			70–79 years			80 + years		
	Men	Women	*p*-value	Men	Women	*p*-value	Men	Women	*p*-value	Men	Women	*p*-value
n	i = 909	*n* = 359		*n* = 250	*n* = 156		*n* = 72	*n* = 62		*n* = 29	*n* = 41	
Poor heath by domains	%	%		%	%		%	%		%	%	
Mobility	4.0	11.9	*p* < 0.001	4.2	17.5	*p* < 0.001	16.0	34.2	*p* = 0.01	39.6	40.2	*p* = 0.96
Self-care	1.7	1.2	*p* = 0.50	2.0	3.5	*p* = 0.33	2.7	7.7	*p* = 0.17	24.7	18.1	*p* = 0.49
Pain/Discomfort	4.1	11.3	*p* < 0.001	7.4	23.6	*p* < 0.001	10.7	25.6	*p* = 0.02	29.0	39.0	*p* = 0.36
Cognition	4.7	7.3	*p* = 0.06	7.3	14.3	*p* = 0.02	13.3	26.1	*p* = 0.06	23.4	33.9	*p* = 0.31
Interpersonal activities	0.8	1.7	*p* = 0.19	1.9	3.1	*p* = 0.42	1.4	6.6	*p* = 0.11	16.7	12.4	*p* = 0.60
Sleep/Energy	2.6	7.5	*p* < 0.001	4.2	14.8	*P* < 0.001	10.7	13.6	*p* = 0.60	25.8	19.8	*p* = 0.54
Affect	1.6	5.7	*p* < 0.001	3.4	11.0	*p* < 0.01	6.6	12.0	*p* = 0.28	17.8	18.5	*p* = 0.94
Vision	2.3	3.0	*p* = 0.43	2.6	7.0	*p* = 0.03	9.4	19.1	*p* = 0.10	25.8	24.6	*p* = 0.91

Note: All analyses weighted to the Nairobi NUHDSS population 2007. *P*-values reported for chi-squared tests of significance comparing men and women for each of the domain

Table 3 shows higher prevalence of poor QoL with older age in both sexes, with women reporting a higher prevalence of poor QoL than men across all age groups. Differences in QoL between men and women were statistically significant (*p* < 0.05) in all age groups, except in the oldest 80-plus years group.

**Table 3:** Prevalence (%) of poor QoL by age and sex, adults aged 50 and over NUHDSS Korogocho and Viwandani Nairobi City, Kenya, 2006–7

	Men	Women	*p*-values	Both Sexes
Total n (%)	1260 (36.4)	618 (55.2)		1878 (42.6)
Age-group n (%)				
50–59 years	909 (32.1)	359 (45.0)	*p* < 0.001	1271 (35.8)
60–69 years	250 (43.6)	156 (64.0)	*p* < 0.001	404 (51.4)
70–79 years	72 (50.7)	62 (77.5)	*p* < 0.01	133 (63.2)
80-plus years	29 (72.3)	41 (77.0)	*p* = 0.64	70 (75.1)

Note: All analyses weighted to the Nairobi NUHDSS population 2007. *P*-values reported for chi-squared tests of significance comparing prevalence between men and women for each age group

**Fig. 1:**
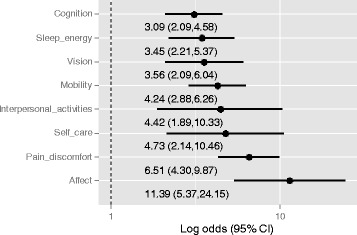
Log odds & 95 % confidence intervals for multivariable logistic regressions showing associations between each domain of function and poor QoL, adults aged 50 and over NUHDSS Korogocho and Viwandani, Nairobi City, Kenya, 2006–7

In separate logistic regressions, domain-specific functional difficulties were statistically significant predictors of poor QoL (*p* < 0.001) after adjusting for possible confounding by socio-demographic factors. Figure 1 shows odds ratios and 95 % confidence intervals for the regression analyses. Notably, respondents reporting problems/difficulties in the affect domain were over eleven times more likely to have poor QoL (OR 11.39: 95 % CI 5.37,24.15) and those reporting problems/difficulties in the pain domain were over six times more likely to have poor QoL (OR 6.51: 95 % CI 4.30,9.87).

Table 4 shows the results of the multivariable logistic regression, including eight indicators of domain-specific function as independent variables in the same regression, and adjusting for socio-demographic factors as possible confounders. The odds of poor QoL among respondents with problems or difficulties for affect, pain/discomfort, mobility and cognition were statistically significant (*p* < 0.05). Although the crude comparisons (Table 1) showed that women were significantly more likely to have poorer QoL than men, this association was not statistically significant in the multivariable model. However older age was significant in association with poor QoL and respondents with relatively less education had statistically significant (*p* < 0.05) higher odds of poor QoL.

**Table 4:** Multivariable logistic regression of functional health and socio-demographic factors associated with poor QoL among adults aged 50 and over NUHDSS Korogocho and Viwandani, Nairobi City, Kenya, 2006–7 (*N* = 1,878)

Functional health: reference good health	Adjusted ORs	(95 % CIs)	*p*-value
Poor function in affect domain	6.95	(3,03–15.96)	*p* < 0.001
Poor function in pain/discomfort domain	3.63	(2.26–5.81)	*p* < 0.001
Poor function in cognition domain	1.83	(1.18–2.85)	*p* = 0.01
Poor function in mobility domain	1.76	(1.11–2.79)	*p* = 0.02
Poor function in vision domain	1.70	(0.90–3.19)	*p* = 0.10
Poor function in self-care domain	1.58	(0.66–3.78)	*p* = 0.30
Poor function in interpersonal activities domain	1.50	(0.53–4.22)	*p* = 0.44
Poor function in sleep/energy domain	1.25	(0.71–2.19)	*p* = 0.45
Sex: reference men			
Women	1.14	(0.87–1.50)	*p* = 0.33
Age-group: reference 50–59 years			
60-69 years	1.46	(1.14–1.86)	*P* < 0.01
70-79 years	1.81	(1.22–2.69)	*P* < 0.01
80 and over	1.80	(0.99–3.30)	*p* = 0.06
Education reference more than 6 years			
Primary <6 years	1.40	(1.04–1.89)	*p* = 0.03
No formal education	1.97	(1.39– 2.80)	*p* < 0.001
Marital status: reference in current partnership			
Now single	1.31	(1.00 – 1.72)	*p* = 0.05
Living arrangement: reference living with other(s)			
Living alone	1.15	(0.84 - 1.57)	*p* = 0.38
Wealth quintiles: reference poorest quintile			
2nd quintile	1.29	(0.93 - 1.79)	*p* = 0.13
3rd quintile	1.30	(0.97 -1.74)	*p* = 0.08
4th quintile	1.07	(0.79 - 1.45)	*p* = 0.66
Least poorest quintile	0.98	(0.72 - 1.32)	*p* = 0.88
Household size: reference 1–2 members			
3-4 members	1.02	(0.74 – 1.40)	*p* = 0.92
5 members and over	1.30	(0.97 – 1.74)	*p* = 0.08

Note: VIF = 1.90; OR = Odds ratio. CI = Confidence Interval. All analyses weighted to the Nairobi NUHDSS population 2007

## Discussion

This study has two sets of important findings. The first is in relation to age and sex-specific perceived difficulties in key domains of functional health, and the second finding refers to multivariable associations between age, sex, domain-specific functions and QoL.

Increasing functional difficulties/problems were reported with advancing age in both sexes and across all domains. The prevalence of functional difficulties/problems was high for mobility, pain/discomfort and cognition in respondents aged 80-plus. Difficulties/problems with self-care were highly prevalent for older men (aged 80-plus). Sex differences in functional health were modified by age. Women aged between 50 and 79 years had significantly poorer self-perceived function than men for mobility and pain/discomfort. In the 50 to 59 year age group, women had significantly more function difficulties than men in the sleep/energy and affect domains. The finding that cognition is highly prevalent is of clinical importance given the increasing rates of dementia in older adult populations worldwide.

The significant relationships we identified between pain/discomfort, cognition and mobility and QoL require further investigation to unravel underlying associations between the domains themselves. We can hypothesise that older adults who endure pain (for example from arthritis) may have lower mobility and a higher risk of falling. Falls can also lead to pain that can have impact on cognition, and poor cognition can also be a risk factor for falls. There are a range of responses and behaviours that can result from chronic pain which we did not investigate here. One current area of public health interest in older populations is that of multi-directional association between pain and alcohol i.e. does pain lead to excess alcohol consumption or does excess alcohol consumption lead to pain and where are falls on this pathway? [30–32]_._ The finding that cognition is highly prevalent is of clinical importance given the increasing rates of dementia in older adult populations worldwide. We did not investigate mental health but this is clearly an important area that can influence functional health.

Given evidence that intrinsic risk factors for falls include older age and female sex [33], targeting women in their 50s and 60s with falls prevention strategies may deliver benefits in this population [30]. While some cognitive decline with advancing age is attributable to biological ageing, cognition limitations impact on the ability of older persons to live independently [34]. Countries such as Kenya face major challenges in planning health and social welfare systems to cater for their ageing populations. These results underscore the need for strategies aimed at attenuating cognitive and mental decline in Nairobi’s urban slum-dwellers [35].

The finding that vision, self-care, interpersonal relations, and sleep were not significant in association with QoL in the multivariable regression, suggests that associations may have been attenuated in the presence of affect, pain/discomfort, cognition and mobility. Vision [36, 37] and sleep, for example, have been shown to be important contributors to older adults’ QoL in LMICs [38, 39].

Quality of life is one of a number of complex components of successful ageing, which might be influenced by different socio-demographic and lifestyle risk factors [40–43]**.** Other research has found that there may be interactions between sex and life-style risk factors such as obesity [42], smoking behaviour [43], and physical activity [40]. Our results show the highest odds of poor QoL in men and women with poor health in the affect domain. This is possibly because ‘affect’ refers to an emotional health state which has bearing on the individual’s well-being and QoL [21, 23, 24]. Strategies aimed at improving QoL in this vulnerable urban population should become public health priorities.

Understanding and unravelling associations between perceived difficulties/problems with function and QoL in older adults can be challenging. Measures of overall self-reported health incorporate complex combinations and interactions between the individual’s assessments of their health. Importantly this study identifies specific functional aspects of health related to poor QoL. These results show that, even after adjusting for potential confounders, difficulties/problems in relation to affect, cognition, mobility and pain/discomfort remain significant in association with poor QoL.

A single overall measure of self-reported health has been widely used in many different studies [44, 45] where it has demonstrated strong predictive power for subsequent morbidity and mortality [44, 46, 47]. However summary measures alone, can mask important aspects of health and functioning. The findings of this study underscore differences in the domains of function that encapsulate the individual’s capacity to perform regular activities. An earlier study of people aged 50 and over in the INDEPTH-WHO SAGE countries reported how the domains of functional health contribute to overall health [48]. The findings from the present study confirm the differential effects of functional health on QoL among people aged 50 and over in Nairobi’s slum settlements. A number of interventions aimed at improving specific aspects of everyday function in this population are feasible. Examples include physical exercise programs to improve mobility, environmental improvements to reduce the risk of falls, and social support networks to improve mental health and cognition [49].

### Strengths and limitations

The domains of function developed by the WHO provide a consistent validated way of describing and comparing population health within and between countries [21–24]. However, cross-country comparison of the results need to be interpreted with caution [41]. Reporting heterogeneity of self-reported functional health across populations in different settings might mislead the comparative findings. The use of anchoring vignettes to identify and adjust for self-rated responses for reporting heterogeneity, has been advocated as a way of addressing this [50]. However the basic assumptions of vignette equivalence and response consistency are not always fulfilled. This can limit the use of anchoring vignettes for cross-country and cross-cultural comparison of self-reported health measures [51].

We acknowledge the possibility of selection bias regarding the sex and socioeconomic composition of the study sample. The study population has an age and sex distribution which is unlike the national population distribution in Kenya but is similar to the distribution in the city of Nairobi city [10]. Kyobutungi et al. [10] noted that urban settlements in Nairobi comprise mainly rural–urban migrants and in general, older females were less likely to migrate from rural areas to cities than older males. This could be because men migrate to urban areas in search of employment, while women are left behind in rural areas to take care of extended families and agriculture [52, 53]. Additionally employment opportunities in urban settlements favour younger males and the older males were relatively disadvantaged in terms of wealth [14]. The results are therefore not generalizable to the wider Kenyan population. Further analyses are needed to validate the findings in this study using other data sets.

The study population is disadvantaged in social and economic terms, being characterized by high levels of poverty and poor living conditions. The wealth quintiles report a relative gradient of wealth (or socioeconomic status) with this population. However we acknowledge that in an environment with high levels of deprivation, such as urban slums, it is possible that differences in wealth are marginal in real terms [10, 13].

The data are cross sectional and describe associations between health and QoL but they do not explain casual relationships. Also, this study is based on self-reported information about health. Future studies are needed to incorporate health examinations and biomarkers within household surveys in order to improve the validity of self-reported health states and to detect and correct systematic reporting biases [22]. Older adults are often defined as those aged 60-plus, but in this study the focus is on older adults aged 50-plus years. Although there can be distinct differences between the two age groups (e.g. in physical and cognitive function, and in regard to employment and pension eligibility) age 50 and above was appropriate here given the short life expectancy in Kenya (62 years). Another limitation is the small number of study participants aged 70 years and over who participated in the study compared to younger categories.

### Public health implications

Past efforts to improve the lives of disadvantaged groups in SSA have focused on infants and children, women of reproductive age, and people with HIV/AIDS [9, 10, 54]. Yet urban slums in Nairobi and other parts of SSA are progressively accommodating disadvantaged older adult first generation rural–urban migrants and their offspring.

Older people in Kenya suffer more poverty compared to the rest of the population and elderly headed households are poorer compared to non-elderly headed households [25]. Nairobi’s urban settlements are characterized by deplorable living conditions including poor housing, lack of clean water, poor sanitation and waste management and inadequate social amenities such as schools and health facilities [26, 55].

Substantial resources are needed to raise the health and living standards of people, particularly the older people in urban settlements in SSA, to come even close to that of their counterparts living in more advantaged circumstances. The persistent gender gap in health, needs to be addressed and closed. Budgets and resources are limited, and ways of measuring specific aspects of functional health, both physical and mental, will become more important in the future with regard to priority setting and resource allocation [22, 56]. Knowledge of different aspects of health, functioning and QoL in older adult slum-dwellers in SSA will provide evidence for governments to set policy priorities, develop infrastructure, implement and evaluate interventions and ultimately assess health policies and programs within strategic frameworks. Differences between the domains of functioning are usually masked by composite measures.  Many older adults suffer poor health in multiple domains. Evidence such as this is important for targeting interventions where they are needed.

Understanding the health and well-being of ageing populations is important for policy and planning, yet, research on ageing and adult health that informs policy predominantly comes from high-income countries. Deficits in everyday functional capacity lend themselves to behaviour change that can promote healthy ageing and enhance QoL. However, behavioural and lifestyle interventions are not common in LMICs, and are more typically accessed by people in high-income countries. This study provides the groundwork for further research of this type. Evidence is needed to improve understanding of associations between functional health and QoL. This can lead to informed development and evaluation of potentially cost-effective interventions suitable for low-resource settings.

## Conclusions

The rapid development of urban slums accommodating increasing numbers of older adults is a serious concern for African governments as they attempt to grapple with the epidemiological transition from communicable to non-communicable diseases. This study highlights a number of areas that can be targeted to improve the physical and psychological health of older-adult slum-dwellers in Nairobi, Kenya. The eight domains of functioning differently impact on QoL. Targeted interventions to improve affect, reduce body pains, enhance cognitive ability and facilitate mobility for older adults should be prioritized. Investing in the health and QoL of older people in SSA will be crucial in helping the region to realise key development goals and open up opportunities for improved health outcomes and viable sustainable economic development.

## Abbreviations

APHRC: African Population and Health Research Centre
HDSS: Health and Demographic Surveillance System
INDEPTH: International Network for the Demographic Evaluation of Populations and their Health
KEMRI: Kenya Medical Research Institute
LMICs: Low- and Middle-Income Countries
NUHDSS: Nairobi Urban Health and Demographic Surveillance System
QoL: Quality of Life
SAGE: WHO’s Study on global AGEing and adult health
SSA: Sub-Saharan Africa
WHO: World Health Organization
WHOQoL: WHO Quality of Life instrument
